# The complete genome sequence and genetic analysis of ΦCA82 a novel uncultured microphage from the turkey gastrointestinal system

**DOI:** 10.1186/1743-422X-8-331

**Published:** 2011-06-29

**Authors:** Laszlo Zsak, J Michael Day, Brian B Oakley, Bruce S Seal

**Affiliations:** 1 Southeast Poultry Research Laboratory, Agricultural Research Service, United States Department of Agriculture, 934 College Station Road, Athens, GA 30605 USA; 2 Poultry Microbiological Safety Research Unit, Agricultural Research Service, United States Department of Agriculture, 950 College Station Road, Athens, GA 30605 USA

**Keywords:** microphage, microviridae, turkey, enteric, metagenomics

## Abstract

The genomic DNA sequence of a novel enteric uncultured microphage, ΦCA82 from a turkey gastrointestinal system was determined utilizing metagenomics techniques. The entire circular, single-stranded nucleotide sequence of the genome was 5,514 nucleotides. The ΦCA82 genome is quite different from other microviruses as indicated by comparisons of nucleotide similarity, predicted protein similarity, and functional classifications. Only three genes showed significant similarity to microviral proteins as determined by local alignments using BLAST analysis. ORF1 encoded a predicted phage F capsid protein that was phylogenetically most similar to the *Microviridae *ΦMH2K member's major coat protein. The ΦCA82 genome also encoded a predicted minor capsid protein (ORF2) and putative replication initiation protein (ORF3) most similar to the microviral bacteriophage SpV4. The distant evolutionary relationship of ΦCA82 suggests that the divergence of this novel turkey microvirus from other microviruses may reflect unique evolutionary pressures encountered within the turkey gastrointestinal system.

## Introduction

Metagenomics analyses have lead to the discovery of a variety of microbial nucleotide sequences from environmental samples [1]. These techniques have also allowed for the discovery of uncultured viral nucleotide sequences that are commonly from bacteriophages [2-4] that has also resulted in the discovery of useful enzymes for molecular biology [5]. There has been a resurgent interest in bacteriophage biology and their use or use of phage gene products as antibacterial agents [6-8]. Bacteriophages are thought to be the most abundant life form as a group [9] and the importance of phage to bacterial evolution [10,11], the role of phage or prophage encoded virulence factors that contribute to bacterial infectious diseases [12-14] and their contribution to horizontal gene transfer [15] cannot be over stated. Additionally, the contribution to microbial ecology [16] and to agricultural production [17,18] is also extremely important.

Enteric diseases are an important economic production problem for the poultry industry worldwide. One of the major economically important enteric diseases for the poultry industry are the poult enteritis complex (PEC) and poult enteritis mortality syndrome (PEMS) in turkeys and a runting-stunting syndrome (RSS) in broiler chickens [19]. Consequently, studies have been ongoing to identify novel enteric viruses among poultry species at our laboratory. In a recent study, we utilized the Roche/454 Life Sciences GS-FLX platform to compile an RNA virus metagenome from turkey flocks experiencing enteric disease [20]. This approach yielded numerous sequences homologous to viruses in the BLAST nr protein database, many of which have not been described in turkeys.

Additionally, we have successfully applied a random PCR-based method for detection of unknown microorganisms from enteric samples of turkeys that resulted in identification of genomic sequences and subsequent determination of the full-length genome from a previously uncultured parvovirus [21]. During these ongoing investigations to further characterize the turkey gut microbiome and identify novel viral pathogens of poultry, bacteriophage genomic sequences have also been identified. Herein we report the complete genomic sequence of a putative novel member of the *Microviridae *obtained from turkey gastrointestinal DNA samples utilizing metagenomics approaches. The protein sequences of ΦCA82 were most similar to those of *Chlamydia *phages.

## Materials and Methods

### Assembly of ΦCA82, a novel member of the *Microviridae *family

Forty-two complete intestinal tracts (from duodenum/pancreas to cloaca, including cecal tonsils) from a turkey farm in California, U.S.A. with histories of enteric disease problems were received at the Southeast Poultry Research Laboratory (SEPRL). The intestines were processed and pooled into a single sample, as previously described [22]. A sequence-independent polymerase chain reaction (PCR) protocol was employed to amplify particle-associated nucleic acid (PAN) present in turkey intestinal homogenates, and has been described elsewhere in detail [22]. Using this approach, a total of 576 clones were identified and sequenced with the M13 forward and reverse primers on an AB-3730 automated DNA sequencer. The sequenced clones were used as query sequences to search the GenBank non-redundant nucleotide and protein databases using the blastn and blastx algorithms [23]. In total, the majority of clones with inserts had no hit in the databases using tblastx [24]. However, 46% of the cloned DNA had homology to cellular DNA, bacterial DNA, bacteriophage DNA, and several eukaryotic viral DNA genomes. Twelve DNA clones had sequence similarity to single-stranded DNA microphages, which have also been identified predominantly in microbialites [25]. A contig, CA82 with an average of eightfold coverage and length of 1962 nt was assembled from eight of those clones. This contig had no significant nucleotide similarity to database sequences, but the deduced amino acid sequence revealed significant similarity to the members of the family *Microviridae*. This initial contig was used to design PCR primers in the opposite orientation of the circular ssDNA to assemble into a contiguous ΦCA82 genome. The PCR amplification resulted in a 3.4 kb product that closed the gap between the CA82 contig and the rest of the circular genome. The final sequence was confirmed by sub-cloning and primer walking with primers resulting ~1 kb fragments containing 250 bp overlapping sequences across the genome. The circular DNA genome was assembled from contigs exhibiting 100% nucleotide identity within the overlapping regions.

### Sequence analysis

The ΦCA82 genome and ORFs were aligned with selected microvirus sequences using ClustalW [26]. Putative ORFs within the ΦCA82 genome were predicted using the FGENESV Trained Pattern/Markov chain-based viral gene prediction method from the Softberry website [27]. Searches for conserved domains within the ΦCA82 genome were performed with the Conserved Domain Database (CDD) Search Service v2.17 at the National Center for Biotechnology Information (NCBI) website [28].

### Comparative genomics of the *Microviridae*

The sequence of phage ΦCA82 was compared to 14 other members of the *Microviridae *(Table 1) obtained from the integrated microbial genomes (IMG) system [29]. To first determine nucleotide level similarities, tetra-nucleotide comparisons between genomes were performed with jspecies [30]. Pairwise genome comparisons were based on regressions of normalized tetra-nucleotide frequency counts and the distributions of the R^2 ^values from these comparisons were visualized in R [31]. To compare genomes based on similarity of predicted gene sequences, the program CD-HIT [32] was used.

**Table 1:** Microviridae sequences used for comparative genomic analyses

Taxonoid	Genome Name	NCBI Taxon ID	Gene Count	Genome Size (bp)	Abbreviation
638275790	Chlamydia phage 1	10857	12	4877	Chp1

638275791	Chlamydia phage 2	105154	8	4563	Chp2

638275792	Chlamydia phage 3	225067	8	4554	Chp3

638275793	Chlamydia phage 4	313629	8	4530	Chp4

638275794	Chlamydia pneumoniae phage CPAR39	117575	7	4532	CPAR39

638275834	Coliphage ID18	338139	11	5486	Col-ID18

638275865	Coliphage WA13	338104	10	6068	Col-WA13

638275985	Enterobacteria phage G4	10843	11	5577	EntG4

645044532	Enterobacteria phage St-1	10845	11	6094	Ent-St1

638276022	Enterobacteria phage alpha3	10849	10	6087	Ent-alpha3

638276027	Enterobacteria phage phiX174	10847	11	5386	X174

638276133	Guinea pig Chlamydia phage	90963	9	4529	CPG1

638276608	Phage phiMH2K	145579	11	4594	MH2K

638276842	Spiroplasma phage 4	10855	9	4421	SpV4

Abbreviations are as shown in Figure 4.

Genomic functional comparisons were based on pfam categories for each predicted gene as classified by the IMG annotation pipeline [29]. A data table of pfam categories and gene counts for each genome was used to construct a similarity matrix and dendrogram in R. To determine which predicted genes were unique to ΦCA82 and those which were shared with other *Microviridae *members, the *Microviridae *pangenome was constructed as the union of all predicted genes from the 14 *Microviridae *genomes and compared to predicted genes for ΦCA82 using both CD-HIT and our data analysis pipeline as described above and blastp run with default parameters except for an e-value cutoff of 0.01.

### Nucleotide accession number

The nucleotide sequence of ΦCA82 genome was deposited in GenBank under accession number HQ264138.

## Results and Discussion

### The ΦCA82 genome

**Figure 1:**
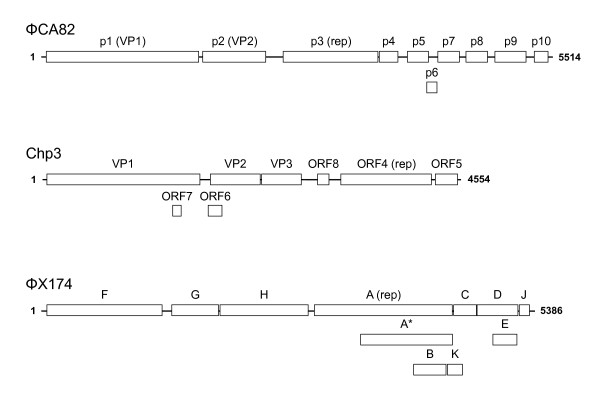
**Comparative graphical representations of the genome organizations for ΦCA82, Chp3, and ΦX174 microviruses**. Computer-predicted open reading frames and the full length genome in nucleotides are indicated. The numbering of nucleotides commences with the start codon of gene 1.

The entire circular, single-stranded nucleotide sequence for the uncultured microvirus ΦCA82 genome was determined to be 5,514 nucleotides. The complete genome sequence had a nucleotide composition of A (38.6%), C (19.6%), G (20.1%), and T (21.6%) with an overall G + C content of 39.7%, which is similar to the chlamydial phages (37-40%). The ΦCA82 genome was organized in a modular arrangement similar to microviruses [33,34] and encoded predicted proteins homologous to those chlamydial bacteriophage types [35] and to the *Bdellovibrio bacteriovorus *ΦMH2K [36]. The coding capacity of the genome is 91% as it encodes ten ORFs, greater than 99 nucleotides similarly to other chlamydial microvirus genomes [35]. The genome size, number of ORFs and total coding % of nucleotides as depicted in Figure 1 is larger than most of the chlamydial phages and is closer in size to the ΦX174 genome [33,34].

### Capsid proteins of ΦCA82

The amino acid identities and homologies between ΦCA82 ORF gene products and predicted proteins from selected phages are presented in Table 2. A total of ten genes could be identified of which only three gene products could be assigned with a known function based upon BLAST analysis. The predicted major capsid protein VP1 encoded by ORF1 belongs to the family of single-stranded bacteriophages and is the major structural component of the virion that may contain as many as 60 copies of the protein [37,38]. The closest sequence similarity of the 565 amino acid ΦCA82 VP1 protein was with the *Spiroplasma *phage 4 (SpV4) capsid protein [39] and the chlamydial phage VP1 proteins [35,38,40-42], as well as the *Chlamydia *prophage CPAR39 [43] and *Bdellovibrio *phage ΦMH2K major capsid protein [36]. A putative minor capsid protein of 234 amino acids was encoded by ORF2 that had similarity to the chlamydial bacteriophages [35,40-43] and the *Bdellovibrio *phage ΦMH2K [36] that was originally postulated to be an attachment protein [38].

**Table 2:** Putative ΦCA82 ORFs and amino acid (aa) homologies with members of *Microviridae*

ΦCA82 ORF	No. of aa	Predicted function	Homologous protein (GenBank accession #)	% amino acid identity (homology)
01	565	major capsid protein	SpV4-VP1 (NC_003438)	30.0 (60.0)
			Chp3-VP1 (NC_008355)	27.0 (63.5)
			CPAR39-VP1 (NC_002180)	28.0 (62.2)
			ΦMH2K-VP1 (NC_002643)	26.0 (58.2)

02	234	minor capsid protein	Chp3-VP2 (NC_008355)	22.0 (45.6)
			ΦMH2K-VP2 (NC_002643)	17.9 (50.8)

03	353	replication initiation	SpV4-rep (NC_003438)	22.6 (54.5)
			ΦMH2K-rep (NC_002643)	17.0 (53.2)

04	71	hypothetical		

05	78	hypothetical		

06	39	hypothetical		

07	80	hypothetical		

08	80	hypothetical		

09	117	hypothetical		

10	52	hypothetical		

^a ^Amino acid identity and homology was determined using ClustalW. Homology was calculated by the sum of identical, strongly similar and weakly similar residues and expressed as percentages of the total.

Recent studies using a comparative metagenomic analysis of viral communities associated with marine and freshwater microbialites indicated that identifiable sequences in these were dominated by single-stranded DNA microphages [25]. Partial sequence analysis of the VP1 gene from these microphages showed that the similarity between metagenomic clones and cultured microphage capsid sequences ranged from 47.5 to 61.2% at the nucleic-acid level and from 37.2 to 69.3% at the protein level, respectively. Interestingly, the VP1 gene of ΦCA82 has a similarly high level of sequence similarity (69.1% at the amino acid level) with the seawater metagenomic phages within the same VP1 region (data not shown). This observation is consistent with an environmental origin of modern poultry phages that have since undergone significant host-specific evolutionary divergence in agricultural settings.

**Figure 2:**
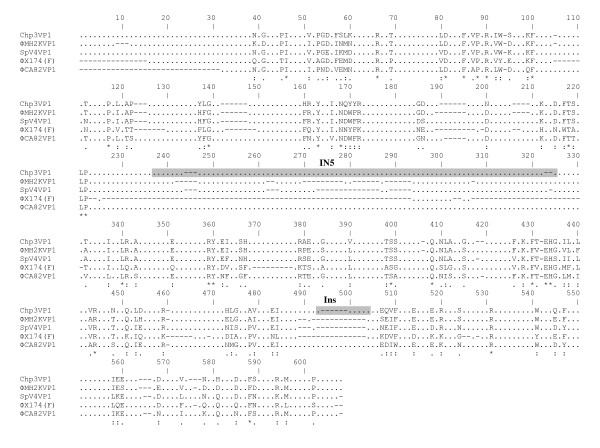
**Multiple amino acid alignment of the major structural protein, VP1 of ΦCA82 with VP1 proteins from other *Microviridae *members**. Deletions (-), non-homologous amino acids (dots in the sequence), identical amino acids (*), residues with strong similarity (:), and weak similarity (.) are indicated. Variable large IN5 and short Ins regions are shaded gray.

A multiple alignment of major capsid proteins among diverse members shows similarities within the entire predicted coding region with the exception of the predicted surface-exposed IN5 loop and Ins (Figure 2). Amino acids located within these regions are involved in forming large protrusions at the threefold icosahedral axes of symmetry in the intracellular microvirus phages [36,41,43]. The IN5 loop, forming a globular protrusion on the virus coat and is the most variable region in the VP1 proteins from *Chlamydia *and *Spiroplasma *phages [44] is potentially located from residues 198 through 295 of ΦCA82 VP1, which is the most highly variable portion of the protein by BLAST. The hydrophobic nature of the cavity at the distal surface of the SpV4 protrusions suggests that this region may function as the receptor-recognition site during host infection. The short variable Ins sequences of ΦCA82 are putatively located from residues 459 through 464 of the VP1 protein.

Chipman et al [44] predicted that the IN5 trimer structure in VP1 may function as a substitute for spike proteins of the ΦX174-like viruses, which are not found in SpV4 or the *Chlamydia *phages, and as such may be responsible for receptor recognition. It has also been suggested that the diverse sequence in this region is associated with host range of phages [36,41,43,44]. The presence of a large insertion in ΦCA82 further supports that it is closer to the intracellular phage subfamily and the sequence dissimilarity within this region between the ΦCA82 and various other phages strongly indicates that this domain indeed may function as a host range determinant.

### Rep protein of ΦCA82

**Figure 3:**
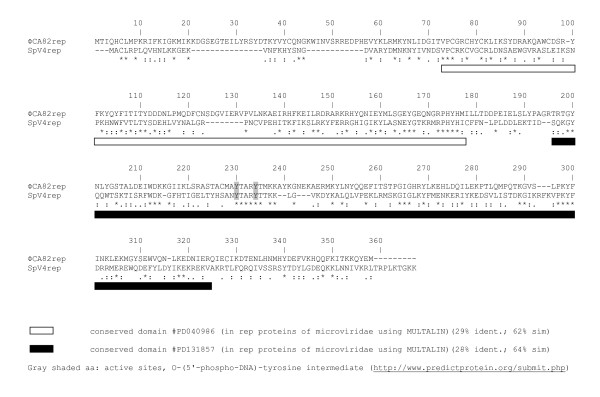
**Pairwise amino acid alignment of the replication initiation protein from ΦCA82 (GenBank accession number **HQ264138**) and SpV4 (GenBank accession number **NC_003438) **microphages**. Identical amino acids (*), residues with strong similarity (:), and weak similarity (.) are shown. Locations of conserved domains are boxed. The active sites of the rep protein are shaded gray.

ORF3 encoded a putative replication initiation protein that was most similar to the SpV4-rep [39] and the *Bdellovibrio *phage ΦMH2K-rep [36] proteins (Table 2). Pairwise alignment of the ΦCA82 VP3 (rep) protein and SpV4 p1 (rep) protein revealed the presence of two conserved domains (Figure 3) from residues 73 through 176 and 195 through 320 of the ΦCA82 protein. Overall, the two rep proteins only had 22.6% identity, but shared many of the same sequences throughout the conserved regions that were recognized by BLAST as putative replication initiation protein regions. Rep protein plays an essential role in viral DNA replication and binds the origin of replication where it cleaves the dsDNA replicative form I (RFI) and becomes covalently bound to it via phosphotyrosine bond (see active sites in Figure 3). The conservation of the functional domains between the ΦCA82 phage rep protein and other microviral replication initiation proteins suggests a similar pathway/mechanism for DNA replication and virion packaging.

### Full genome comparisons of ΦCA82 with other members of the *Microviridae*

**Figure 4:**
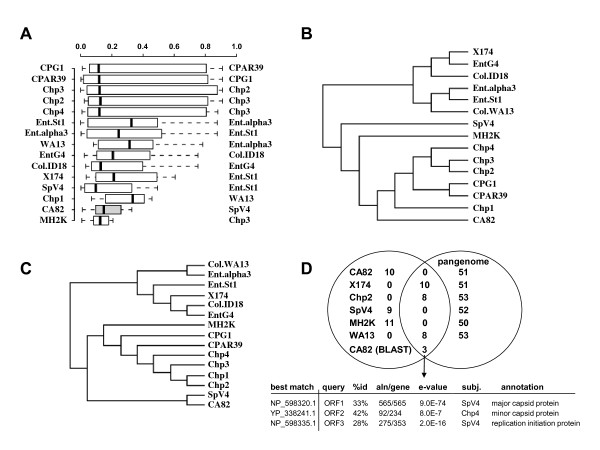
**Genome-based comparisons of ΦCA82 and 14 other *Microviridae***. A) Comparisons of tetra-nucleotide frequencies as a measure of genome similarity at the nucleotide level. Boxplot represents the distributions of R^2 ^values for pairwise comparisons of tetra-nucleotide frequencies for each genome compared to all others. For each genome, the closest match (highest R^2^) is shown to the right. B) Genomic sequence similarity as assessed by clustering of predicted amino-acid sequences. Clustering was completed with CD-HIT as described in the text, which accomplishes an all-versus-all comparison of each predicted protein. Sequence clusters were determined with a 40% similarity cutoff (sequences must be >40% similar for membership within the same cluster); tree is based on cluster memberships across all genomes. C) Genomic functional similarity as assessed by gene counts for each genome in pfam categories as classified by IMG. Tree construction methodology is essentially identical to (B), but based on functional cluster membership rather than clustering based on sequence similarity. D) Shared and unique predicted genes between ΦCA82 and all 14 other *Microviridae *members as determined by the CD-HIT clustering used for (B) and BLAST as described in the text.

The ΦCA82 genome is quite different from other members of *Microviridae *as indicated by comparisons of nucleotide similarity, predicted protein similarity, and functional classifications. Comparisons of ΦCA82 to 14 other *Microviridae *genomes showed very low correlations of tetra-nucleotide frequencies as a measure of genome similarity. ΦCA82 was most similar to SpV4, but the correlation of tetra-nucleotide frequencies was poor (R^2 ^= 0.33; Figure 4A). Only ΦMH2K had lower similarities to other *Microviridae *(Figure 4A). Clustering of predicted proteins showed ΦCA82 was most closely related to a clade comprised of the chlamydial phages, but as in the nucleotide comparisons, the predicted proteins of ΦCA82 are quite distinct from those of the other microviruses (Figure 4B). Function-based clustering of genomes using pfam categories showed that ΦCA82 was most similar to SpV4 (Figure 4C), based on shared membership of the ΦCA82 ORF1 in pfam02305, an F super family capsid protein. These results were confirmed by comparisons of predicted proteins from the ΦCA82 genome to a *Microviridae *pangenome. This analysis showed only three genes with significant similarity as determined by local alignments using blastp with no overlap between ΦCA82 and the *Microviridae *pangenome based on global alignments at a 40% similarity cutoff (Figure 4D). ΦCA82 is only distantly related to other *Microviridae*, but is most similar to SpV4 and the chlamydial phages. In summary, the whole genome comparisons of ΦCA82 to other *Microviridae *members indicate a distant evolutionary relationship, perhaps suggesting that the divergence of ΦCA82 from other microviruses reflects unique evolutionary pressures encountered within the turkey gastrointestinal system.

## Competing interests

The authors declare that they have no competing interests.

## Authors' contributions

LZ conceived and coordinated the study, performed the PCR and sequencing, and wrote the paper. JMD prepared the samples and performed bioinformatic analyses. BBO coordinated the bioinformatic analyses. BSS provided technical input and intellectually contributed to the study design and writing the paper. All authors read and approved the final manuscript.
